# Large Scale Comparison of Innate Responses to Viral and Bacterial Pathogens in Mouse and Macaque

**DOI:** 10.1371/journal.pone.0022401

**Published:** 2011-07-18

**Authors:** Guy Zinman, Rachel Brower-Sinning, Chineye H. Emeche, Jason Ernst, Grace Tzu-Wei Huang, Shaun Mahony, Amy J. Myers, Dawn M. O'Dee, JoAnne L. Flynn, Gerard J. Nau, Ted M. Ross, Russell D. Salter, Panayiotis V. Benos, Ziv Bar Joseph, Penelope A. Morel

**Affiliations:** 1 Lane Center for Computational Biology, Carnegie Mellon University, Pittsburgh, Pennsylvania, United States of America; 2 Department of Computational Biology, University of Pittsburgh, Pittsburgh, Pennsylvania, United States of America; 3 Department of Microbiology and Molecular Genetics, University of Pittsburgh, Pittsburgh, Pennsylvania, United States of America; 4 Department of Immunology, University of Pittsburgh, Pittsburgh, Pennsylvania, United States of America; Kyushu Institute of Technology, Japan

## Abstract

Viral and bacterial infections of the lower respiratory tract are major causes of morbidity and mortality worldwide. Alveolar macrophages line the alveolar spaces and are the first cells of the immune system to respond to invading pathogens. To determine the similarities and differences between the responses of mice and macaques to invading pathogens we profiled alveolar macrophages from these species following infection with two viral (PR8 and Fuj/02 influenza A) and two bacterial (*Mycobacterium tuberculosis* and *Francisella tularensis* Schu S4) pathogens. Cells were collected at 6 time points following each infection and expression profiles were compared across and between species. Our analyses identified a core set of genes, activated in both species and across all pathogens that were predominantly part of the interferon response pathway. In addition, we identified similarities across species in the way innate immune cells respond to lethal versus non-lethal pathogens. On the other hand we also found several species and pathogen specific response patterns. These results provide new insights into mechanisms by which the innate immune system responds to, and interacts with, invading pathogens.

## Introduction

Lower respiratory tract infections are the single largest cause of death in low income countries and the fourth largest cause of death in middle and high income countries [1], [2]. The infections may be caused by bacteria (e.g., *Mycobacterium tuberculosis*) or viruses (e.g., influenza). Influenza virus infects 5–10% of the world's population each year and results in approximately 500,000 deaths annually [3]. A third of the world's population is thought to be infected with *Mycobacterium tuberculosis* (Mtb) [2]. In addition, the emergence of pathogens that may be released through acts of terrorism, such as *Francisella tularensis* (Ft), creates new public health concerns [4]. While therapies and vaccines exist for many of these infections there is a need to increase our understanding of the early host response to these infections. For example, when new influenza viruses appear each year due to antigenic variation, the early immune response can either be excessive leading to increased morbidity and mortality, or be inadequate thereby allowing the pathogen to spread beyond the confines of the lung [5]. Comparing and contrasting the early host responses to diverse pathogens will provide valuable insight into the pathogenesis of emerging and biodefense pathogens.

Experimental animal models are commonly used to study the natural history of specific infections and the response to vaccines. The murine model is an attractive model used in studies of influenza [6] and Mtb [7] infection due to the wide availability of immune reagents and genetically altered mouse strains. The mouse model has drawbacks, however, since the pathogenesis of infections may not fully recapitulate human infection. The non human primate, such as the macaque, has emerged as a model that may more closely resemble human disease in studies of both influenza [8] and Mtb [9], [10]. Our study examines the ability of alveolar macrophages (AM) obtained from mice and macaques to respond to the same pathogens. This provides a unique opportunity to compare responses to important pulmonary pathogens both within, and between, species that are relevant to human disease.

AM are the first cells of the innate immune system to interact with pathogens invading the lung [11]. These cells line the alveolar spaces and, in the steady state, maintain an anti-inflammatory environment in the lung, maximizing gas exchange between airspaces and the bloodstream [11]. This anti-inflammatory environment is promoted by cytokines such as TGF-β and cell surface molecules such as CD200/CD200R [12], [13]. When pathogens enter the lower airways, AM respond to the presence of molecules derived from pathogens, as well as cytokines or molecules associated with cellular damage that may be secreted by infected epithelial cells. Previous studies have shown that pathogens can ensure survival either by thwarting activation of AM [14] or by inducing an over-exuberant response, which may lead to immunopathology [15]. Infection with some pandemic influenza strains can induce an intense inflammatory response [8]) leading to increased morbidity and mortality. In contrast, infection with virulent bacteria such as Ft results in low levels of inflammatory cytokine production, allowing the bacteria to escape immune-mediated destruction and disseminate systemically [16]–[19].

A better understanding of how AM respond to a wide array of pathogens is important for the design of novel therapeutic agents that might be used to treat potentially lethal pulmonary infections. To assess the different strategies AMs use to respond to infectious agents, we performed a large scale study to compare responses to both bacterial and viral pathogens, in two species; C57BL/6 mouse and cynomolgus macaque *(Macaca fascicularis)*. The goal of this study was to collect a large dataset profiling the dynamics of the responses in these two species. As we show, such datasets can serve as a resource for studying similarities and differences between innate immune responses both within and between the two species.

We examined responses, using Luminex and microarrays, to the bacteria Mtb and Ft subspecies *tularensis*, strain Schu S4. (Schu S4) and two influenza A viruses, A/Fujian/411/2002(H3N2) (Fuj/02) and the mouse adapted A/PR8/34(H1N1) (PR8). Mtb causes a chronic infection characterized by granulomatous inflammation and progressive pathology in the lungs of mice, whereas macaques infected with a low dose of Mtb either develop active or latent TB in a manner very similar to human infection [9], [20]. Fuj/02 is a human influenza virus that causes a productive infection in mice and causes only mild infection in macaques, whereas PR8 causes a lethal infection in mice and only mild infection in macaques. *In vivo* infection with Schu S4 is rapidly lethal in mice and has around 30% mortality in macaques. These studies provide a comprehensive analysis of the response of a key cell type in the pulmonary innate immune system and identify differences and similarities between the responses to acute and chronic infections.

## Results

### Characterization of alveolar macrophages from mice and macaques

AM isolated from C57BL/6 mice and cynomolgus macaques by bronchoalveolar lavage (BAL) were CD11c^+^ CD11b^−/lo^ and negative for CD86. In addition, mouse AM were F4/80^+^, expressed low levels of MHC class II and were positive for CD80 (Figure S1). Despite the expression of CD11c, which is often considered a dendritic cell marker, the cells had the histological appearance of macrophages (Figure S2A).

Murine AM were exposed to a series of TLR ligands to characterize their responsiveness to defined stimuli. Exposure of murine AM to poly I∶C and LPS led to marked upregulation of CD86 expression (Figure S2B). The observed pattern of cytokine production was indicative of activation of the MyD88/NF-κB (LPS), TRIF/IRF3 (LPS and poly I∶C) and TRIF/IRF7 (poly I∶C) pathways in these cells [21] (Figure S2B, S2C). No cytokines were detected following exposure to medium alone or CpG, consistent with the observation that murine AM do not express TLR9 [22]. Exposure of macaque AM to LPS or poly I∶C failed to induce upregulation of CD86 but the cells secreted CCL4, IL-6 and TNF in response to both LPS and poly I∶C (data not shown).

### Murine and macaque AM show distinctive responses to bacteria and virus

**Figure 1:**
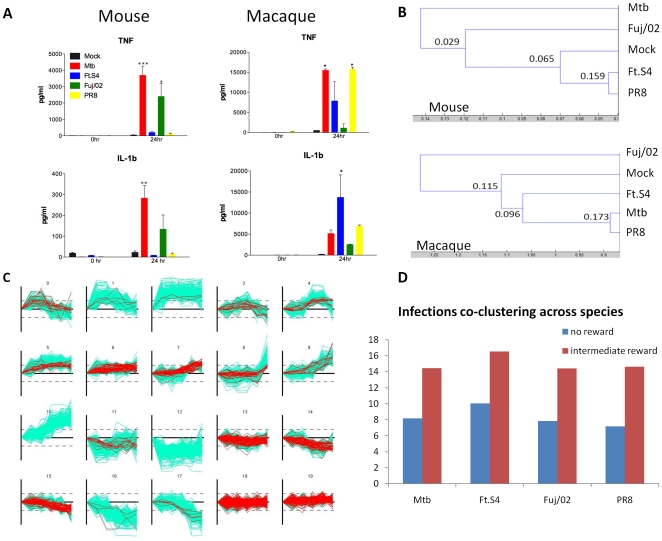
Pathogens that give rise to lethal infections *in vivo* elicit weak responses from AM. (A) AM from C57BL/6 mice or cynomolgus macaques were exposed to the indicated pathogens. Supernatants were collected at 0 hr and 24 hr following exposure and examined for the production of TNF and IL-1β by Luminex as described in the Methods. Results are expressed as the mean ± SEM of 3–8 independent experiments. Statistics were performed using one way ANOVA with a Tukey post test. P values: * <0.05, ** <0.01, *** <0.001. (B) Hierarchical Clustering of differentially expressed (DE) genes from murine (1383 genes) and macaque (1387 genes) AM following exposure to the indicated pathogens. The dendrograms were generated as described in Methods. The numbers in the dendrograms represent the frequency with which a particular node was seen in 1000 permutations of the algorithm and gives a level of confidence in the observed structure. (C) Resulting clusters of Softclust analysis over DE genes from all infections in both species. Marked in red are genes that changed cluster assignment following an intermediate orthology reward compared to no-reward run. (D) Infections co-clustering across species in no-reward and intermediate reward settings. Co-clustering increased 82% on average in the rewarded run.

For a comprehensive assessment of the cytokine response of AM to each pathogen, isolated cells were first allowed to settle in the culture well for 30 minutes prior to infection. Cell lysates and SN were collected at time 0, 1, 2, 6, 12, and 24 hours post infection. Cytokines were detected as early as 2 hours post infection (Figure S3) and these peaked at 6 hours and were maintained at the same levels until 24 hours post infection (data not shown). In mice, significant levels of TNF and IL-1β were observed following infection with Mtb and Fuj/02 influenza, whereas no cytokines were detected following exposure to PR8 or Schu S4 (Figure 1A). In macaques, Mtb and PR8 influenza induced strong TNF production, whereas Schu S4 induced IL-1β (Figure 1A). No cytokines were observed following Fuj/02 influenza exposure in macaques.

### Profiling responses using microarrays

To further characterize the sets of genes involved in innate immune response in these two species we performed microarray analysis on RNA samples collected from these experiments. A total of 217 arrays (Table 1, EBI Array Express accession number E-MTAB-427) were hybridized for the 6 time points, however, no RNA samples were analyzed for the 24 hour time point from mock-infected or PR8-infected murine AMs due to poor viability of the cells by that time point.

**Table 1:** Number of arrays used in the analysis.

		0 hr	1 hr	2 hrs	6 hrs	12 hrs	24 hrs	Total
Mouse	**Mock**	12	3	3	4	2	0	24
	**Mtb**	4	4	4	2	2	4	20
	**Schu S4**	8	6	8	5	5	6	38
	**Fuj/02**	6	4	4	4	2	2	22
	**PR8**	3	2	3	3	2	0	13
Macaque	**Mock**	3	3	3	3	3	2	17
	**Mtb**	4	3	4	4	4	3	22
	**Schu S4**	3	3	5	5	6	4	26
	**Fuj/02**	2	3	3	2	5	1	16
	**PR8**	3	2	3	4	2	1	15
Total								**217**

Following normalization and log transformation differentially expressed (DE) genes were selected based on two criteria. 1) *p*-values were assigned to each gene by calculating a two sided t-test between the time point series and the replicates of time point 0 for that gene and genes with a *p*-value above a cutoff of the 10% quantile for the array were filtered out. 2) the list of genes was further trimmed down by selecting only genes whose absolute log_2_ fold change compared to time point 0 exceeded a cutoff value of 2.5 in at least two time points. Using these criteria a total of 1383 mouse and 1387 macaque DE genes were identified (Table S1). A full list of these genes with expression values can be found in Tables S2 and S3.

The global similarities between the sets of DE genes for each species/infection were analyzed by performing a dendrogram analysis (Figure 1B). The dendrograms were produced using hierarchical clustering of DE genes as described in the Methods. No significant tree topology was identified for the macaque datasets. This appears to be related to the higher degree of activation observed in the mock infection, which may be due to the fact that these animals are exposed to more inflammatory stimuli in their housing and environment. However, it appears that the responses elicited by PR8 influenza, Schu S4, and Mtb are more closely related to each other than to mock or Fuj/02 influenza infection (Figure 1B), which correlates with the observed pattern of cytokine production (Figure 1A). Similar analysis of the mouse datasets reveals that Schu S4, PR8, and mock-infected cells cluster together (p-value = 0.065). Mtb infected cells induced a distinct response (p-value 0.029) (Figure 1B). A more detailed global view on similarities and differences across species was obtained using SoftClust to cluster genes in both species across all pathogens [23]. Like *k*-means SoftClust attempts to maximize a target function that is based on the agreement of genes with the clusters they are assigned to by modifying the cluster centers in an iterative manner. However, SoftClust also tries to maximize the assignment of orthologs to the same cluster allowing it to overcome problems related to noise and arbitrary boundary of clusters. This weight guarantees that orthologs are assigned to same cluster unless there is a strong evidence to separate them. We used SoftClust to cluster all data in this study. The conservation rates for the different infections across species with no reward are ∼8% on average and under the intermediate orthology reward value the conservation rates are ∼15% on average (Figure 1D) with insignificant increase in cluster variance (Figure 1C). General clustering trends for DE genes show that orthologs are clustered 4 times more within species than between pathogens highlighting the differences between the infections progression in the two species and the need for cross species study. Examining co-clustering of infections and mock arrays we found out that mouse PR8 influenza is clustered 24% more with the mock arrays compared to the other infections, consistent with the lack of cytokine production observed (Figure 1A). Figure 1C presents the 20 resulting clusters obtained under an intermediate orthology reward. Each cluster contains genes from both species and from all pathogens. Cluster 2, which is upregulated early in the response, is enriched for inflammatory response (corrected p-value of 10^−18^), immune response, and chemokine activity for all macaque infections and macrophage differentiation for viral infections. Cluster 9 is enriched for immune response for bacterial infections in both macaque and mouse (corrected p-values of 10^−35^ and 10^−12^ respectively), but viral infections are enriched for immune response only in macaque (10^−32^).

### Identification of a core set of immune response genes

**Figure 2:**
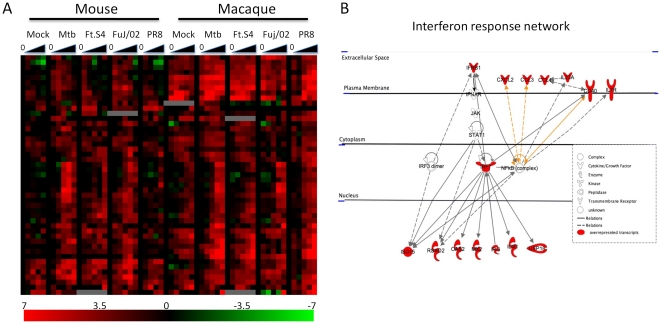
A core set of immune-related genes are upregulated in AM from both mouse and macaque. (A) Heatmap of the 47 DE genes that are upregulated in both mouse and macaque. The shows the time course of DE gene expression in AM exposed to medium (mock), Mtb (TB), Schu S4 (Ft.S4), influenza Fuj/02 or PR8. The time points analyzed were 0, 1, 2, 6, 12 and 24 hours post infection, and the values shown represent the median of all replicates at a particular time point. The scale represents fold change from time point 0 of −7–+7 (log_2_). (B) Pathway analysis of the 47 upregulated genes using Ingenuity software revealed significant overrepresentation of genes from the interferon response pathway, which is depicted here.

**Figure 3:**
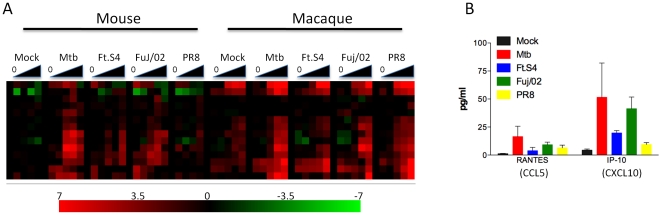
Mtb infection of AM induces robust activation of the interferon pathway. (A) Heatmap showing the expression profile of interferon response genes in murine and macaque AM exposed to the indicated pathogens. Infection with Mtb induces the most robust and sustained expression in both mouse and macaque. The scale represents fold change from time point 0 of −7–+7 (log_2_). (B) Luminex analysis of supernatants from murine AM exposed to the indicated pathogens demonstrating the production of the interferon induced chemokines, CCL5 and CXCL10 following Mtb infection. Results shown are the mean ± SEM of 2–8 independent experiments.

Examination of the almost 1400 DE genes in murine and macaque AM revealed a core set of 91 genes that were DE in both species. Of these, 47 were upregulated in all infections and across both species (Figure 2A). These genes are likely ‘core’ immune response genes as they are activated in all types of infections studied and are conserved across the two species (Figure 2A). Gene ontology (GO) analysis for these genes validated this proposition, since this set of genes was enriched for cytokine activity (corrected *p* value, *p*_corr_ = 10^−11^) and immune response (*p*_corr_ = 10^−7^). Pathway analysis software from Ingenuity Systems was used to identify the networks associated with these genes (Figure 2B). Canonical pathways significantly over-represented among these genes are shown in Table 2. Of note, the response to virus pathway is prominent among these genes and included such genes as IFN-β, IL-23a and IRF7. This is perhaps not surprising for the influenza virus infections, but Mtb infection seemed to induce the most robust activation of this pathway (Figure 3A). In addition, the secretion of the interferon-induced chemokines CCL5 and CXCL10 were induced following Mtb infection of murine AM (Figure 3B). These results suggest that Mtb is able to activate the interferon pathway in AM.

**Table 2:** Canonical pathways over represented in the genes that are upregulated in the core set of differentially expressed genes.

Pathway	−log (B-H p-value)	Ratio	Molecules
Hepatic Fibrosis/Hepatic Stellate Cell Activation	5.32	0.05	TIMP1, IL1A, CD40, CCL5, CSF1, IL6, MET
Communication between Innate and Adaptive Immune Cells	5.29	0.06	CCL4, IL1A, CD40, IFNB1, CCL5, CCL3
Role of Cytokines in Mediating Communication between Immune Cells	4.78	0.09	IL1A, IFNB1, CSF3, IL6, IL23A
Activation of IRF by Cytosolic Pattern Recognition Receptors	4.55	0.07	CD40, IFNB1, ISG15, IL6, IRF7
Pathogenesis of Multiple Sclerosis	4.51	0.33	CCL4, CCL5, CCL3
Dendritic Cell Maturation	2.91	0.27	IL1A, CD40, IFNB1, IL6, IL23A

### AM response to Mtb infection is species-specific

**Figure 4:**
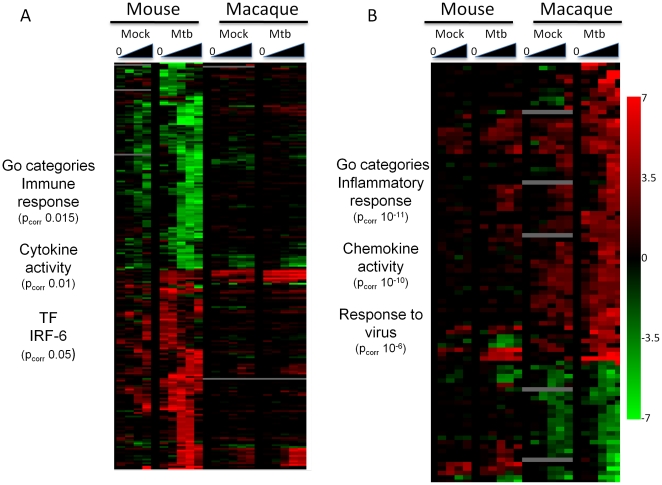
Response of AM to Mtb is species-specific. (A) Heatmap of genes identified as DE by infection of murine AM with Mtb. Also plotted are the expression values for the ortholog macaque genes following Mtb infection, and following mock infection. (B) Heatmap of genes identified as DE by infection of macaque AM with Mtb. Also plotted are the expression values for the ortholog murine genes following Mtb infection. Overrepresented GO categories are shown next to each heatmap. The scale represents fold change from time point 0 of −7–+7 (log_2_).

AM from both mouse and macaque showed a robust cytokine response when infected with Mtb, and the gene expression profiles were examined for similarities between these species. A total of 695 genes were DE in macaque Mtb infection while 429 genes were DE following Mtb infection of murine AM (Table S1). Analysis of the GO categories over-represented following Mtb infection of murine AM revealed over-representation of genes associated with the immune response (p_corr_ = 0.015) and cytokine activity (p_corr_ = 0.01). When the expression values for genes DE following Mtb infection of murine AM were plotted along with values for known orthologs in macaque only a small number of genes were seen to have a similar behavior (Figure 4A). The majority of orthologous macaque genes were not altered following Mtb infection. A similar phenomenon was observed when genes DE following Mtb infection of macaque AM were plotted alongside the murine orthologs (Figure 4B). Again, only a minority of these genes showed a similar pattern in Mtb-infected murine AM. Over-represented GO categories in the macaque Mtb set included the inflammatory response (p_corr_ 10^−11^), chemokine activity (p_corr_ 10^−10^) and response to virus (p_corr_ 10^−6^). Thus, while Mtb infection of AM from both species induces immune response-related genes these are, for the most part, non-overlapping.

### Divergent response to influenza A virus between species

**Figure 5:**
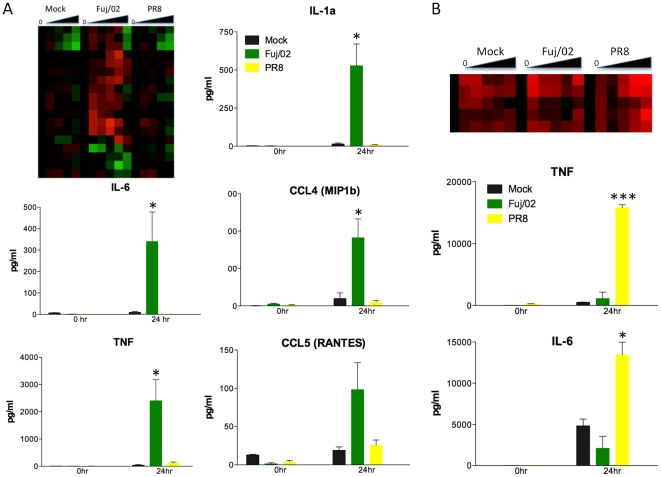
AM do not respond to exposure lethal influenza A flu viruses. (A) Heatmap of immune response gene expression profile of murine AM exposed to Fuj/02 or PR8. The response elicited by PR8 is indistinguishable from that seen following mock infection. Luminex analysis of cytokines (IL-1α, IL-6 and TNF) and chemokines (CCL4 and CCL5) present in supernatants collected at 0 and 24 hours post exposure to Fuj/02 and PR8. No cytokines are detected in SNs from cells exposed to PR8 but robust production is seen when cells are exposed to Fuj/02. Results shown are mean ± SE of 3–8 independent experiments. (B) Heatmap of cytokine and chemokines gene expression profile of macaque AM exposed to Fuj/02 or PR8. Both mock and Fuj/02 infection exhibit transient activation of these cytokines, whereas PR8 exposure induces sustained expression. Luminex analysis of TNF and IL-6 levels confirms the increased production cytokine following PR8 exposure. Results shown are mean ± SEM of 3 independent experiments. Statistics were performed using one-way ANOVA with a Tukey post test. P values * <0.05, *** <0.001.

Influenza PR8 induces a lethal infection in mice, but does not productively infect macaques. Analysis of the cytokines produced by murine AM exposed to PR8 influenza suggested that these cells failed to respond to this challenge (Figure 1A). In contrast, AM from macaques responded to this virus, but showed a reduced response to Fuj/02 influenza. Examination of the gene expression profiles in response to the two influenza viruses confirmed this observation (Figure 5). Murine AM increased gene expression of several cytokines in response to Fuj/02 influenza, but the response to PR8 influenza was almost identical to that elicited by mock infection (Figure 5A). Luminex analysis of these cytokines and chemokines confirmed the observed gene expression pattern. The opposite pattern was seen in AM from macaque with increased and sustained levels of cytokine gene expression observed in AM exposed to PR8 influenza, whereas the response following exposure to Fuj/02 influenza was transient and not sustained, similar to mock infection (Figure 5B). This gene expression translated into robust cytokine production following exposure to PR8 and little or no cytokine production in response to Fuj/02 influenza (Figure 5B).

### The transcription factor IRF7 coordinates different gene expression patterns depending on the infection

**Figure 6:**
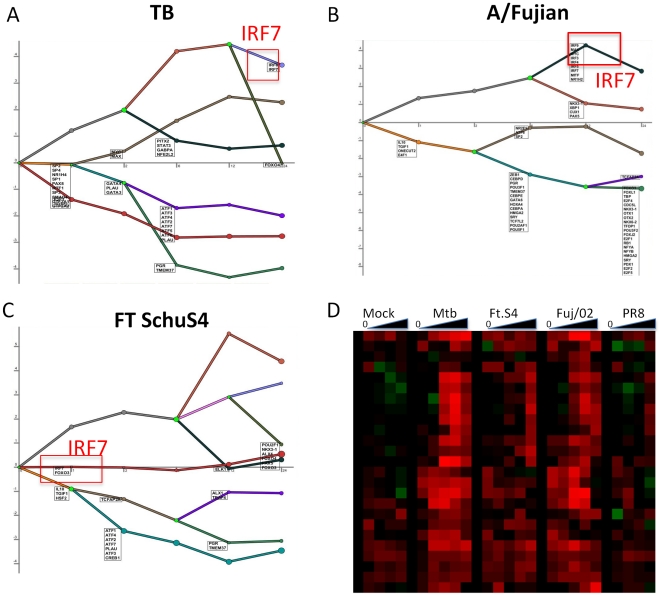
Analysis of transcription factors reveals a changing pattern for IRF7 following infection of murine AM. (A, B and C). Analysis of the transcription factors responsible for different pattern of gene expression using the program DREM revealed divergent roles for IRF7 in Mtb (A) Fuj/02 (B) and Schu S4 (C) infection of murine AM. The program identifies transcription factors responsible for bifurcations and gene trajectories and these are listed at the appropriate in order of significance. The position of IRF7 is highlighted with the red square. Lines represent the average expression patterns of DE genes for each of the infections shown. D. Heatmap of genes known to be controlled by IRF7 that were included in the DREM analysis, confirming the divergent expression patterns of these genes in the three infections.

In addition to cross species analysis the expression data we collected enabled us to study the regulatory networks involved in divergent responses to pathogens within an individual species. We used the program DREM [24], which reconstructs dynamic regulatory networks and identifies transcription factors responsible for unique expression patterns. DREM identified distinct patterns of transcription factor activation in murine AM infected with Mtb, Schu S4 and Fuj/02. In particular IRF7 was identified as a transcription factor controlling the up-regulation of genes following exposure to Mtb and Fuj/02 influenza, whereas genes controlled by this transcription factor only increased late in infection with Schu S4 (Figure 6A–C). Heat map plots of IRF7-controlled genes reveal the divergent responses to the three infections (Figure 6D). These genes include IRF7, IL-6, IFN-β, IKK-ε, mx2, rsad2, oas1 and oas3. Many of these genes are interferon-inducible [25]. This analysis confirms the finding that Mtb infection of murine AM induces a type 1 interferon pathway similar to that seen induced by the influenza virus Fuj/02 influenza (Figure 6D). Schu S4 infection failed to induce this gene expression pattern, although expression of several of the genes appeared to increase expression at the later time points. Again, the pattern for PR8 influenza was almost indistinguishable from the mock infection.

## Discussion

Genes are highly conserved between close species allowing the study of biological systems, and several diseases, using model organisms. Several human immune response genes have been identified based on studies in mice and other species. However, while sequence and overall function of genes are highly conserved, in several cases responses to the same pathogens, or to drugs, differs between humans and mice. Thus, in order to study the similarities and differences of the innate immune response system we need to supplement sequence data with functional genomics data.

### Similarities and differences in the innate immune response between mice and macaques

In this paper, we present a resource that will allow researchers to perform such comparisons, both within and between species. To facilitate within species studies we looked at responses to both bacteria and viruses. To allow for cross species comparisons, we studied both mice and macaques. For these, we studied the response of an important cell of the pulmonary innate immune system, the alveolar macrophage (AM), to infections. We identified a core set of genes that was upregulated in AM from both mice and macaque and are implicated in the response to virus pathway. We also observed that, in both species, pathogens that cause lethal infection induced weak or absent responses in exposed AM. This suggests that blunted induction of responses in AM is correlated with an inability to control infection, indicating the importance of the innate response in the airways for initial control of infection by pathogens. Infection of murine or macaque AM with Mtb induced robust cytokine production but the patterns of gene expression changes in the two species were largely non-overlapping. Divergent responses to two influenza A viruses were observed in mice and macaque AM, such that murine AM failed to respond to the lethal PR8 influenza virus, whereas macaque AM responded robustly to this virus, but weakly to Fuj/02 influenza. The response of murine AM to the Schu S4 appeared to be aborted, whereas macaque AM responded vigorously to this challenge. These results provide a global view of the innate immune response to varied pathogens and highlight some important species differences.

### Lethal influenza viruses do not induce strong response in AM

AM play important roles in controlling viral replication [5], [26] and limiting immunopathology [5], [13] following acute infection with influenza viruses. In the case of influenza, depletion of macrophages prior to infection leads to uncontrolled viral replication [27] and a recent study has implicated the chemokine CCL2 (MCP-1) in alveolar regeneration following influenza infection. Mice treated with anti-CCL2 mAb had reduced cellular infiltration, including macrophages and neutrophils, following influenza infection [28], further implicating the role of macrophages in the resolution phase of the infection. The PR8 influenza virus used in this study was a mouse-adapted strain that causes severe morbidity and mortality in mice, but causes minimal disease in macaques or humans. In *vitro* exposure of murine AM to PR8 influenza failed to induce a cytokine response. Analysis of the gene expression profile demonstrated that the pattern induced by PR8 influenza was indistinguishable from mock-infected AM. This effect was specific to murine AM since macaques AM were able to mount a robust response, as detected by cytokine production and gene expression profiling. A previous study [29] analyzed the ability of several influenza A viruses to infect murine macrophages. Results showed that PR8 had the lowest rate of infectivity and this was correlated with a reduced ability to bind to the mannose receptor [29]. This suggests that one of the adaptations acquired by PR8 is a reduced ability to be taken up by AM, thereby avoiding the induction of a robust innate response. In contrast *in vivo* infection with PR8 influenza does induce cytokine/chemokines production in the lung [30], [31] and this is likely due to direct infection of airway epithelial cells, leading indirectly to macrophage activation [32]. The human Fuj/02 influenza virus does not cause disease in mice and murine AM responded vigorously to this virus. While the Fuj/02 influenza virus does not cause a lethal infection in macaques we did observe a reduced response to this virus in macaque AM. Overall these results suggest that the more lethal influenza viruses have evolved to avoid either being taken up by AM, or inducing a strong inflammatory response, thereby allowing the virus to replicate and cause severe symptoms.

### Schu S4 employs active mechanisms to abort activation of infected AM

Schu S4 infection of murine AM failed to induce a robust cytokine response as previously reported [14], [33]. It has been shown that Schu S4 productively infects macrophages and escapes from the phagosome into the cytoplasm where it rapidly proliferates and kills the cell [34]. We have observed that the doubling time for Schu S4 is around 3 hours in murine macrophages. We observed two distinct gene expression patterns in murine AM infected with Schu S4. In the first, immune response genes such as IL-6, IL-1α, IL-1β and IFN-β were upregulated one hour following infection but this upregulation rapidly returned to baseline and was not sustained. In contrast, the expression of some genes under the control of the IRF7 transcription factor, including IRF7, IKK-ε, mx2, rsad2, oas1 and oas3, was delayed compared to other infections and only observed late in the course of the infection. These results suggest that Schu S4 employs active mechanisms to abort activation of infected AM, leading to a blunted innate immune response to this lethal pathogen. Again AM from macaques did respond to infection with Schu S4 as seen both by the levels of cytokines produced and the pattern of gene expression. Few studies have examined Schu S4 infection in cynomolgus macaques but the few reports that exist suggest that it is a serious infection with a high level of mortality [35]. The more robust response to Schu S4 infection by macaque AM could be related to the general immune status of macaques as evidenced by the overall increased activation seen in the mock infected AM. Unlike the mice, which were housed in microisolator cages, the macaques used in this study were kept in an environment that exposed them to normal air, and therefore potentially to microorganisms. This exposure could have altered the ability of AM to respond to Schu S4 infection, a concept that was recently reviewed [36] and is potentially more similar to the human situation. Human monocyte derived macrophages have a limited response to Ft [18], [19], suggesting important differences in immunological responses could be observed depending on the source of macrophages as well as host species.

### A core set of genes upregulated in both murine and macaques in response to Mtb infection

While AM from mice and macaque showed divergent responses to influenza A virus and Schu S4, Mtb infection of AM induced strong cytokine response in both species. This infection therefore provided a useful opportunity to analyze the similarities and/or differences between these two species. Our own experience with Mtb infection in macaques suggests that the macaque model is much closer to human TB than the mouse model [9], [20]. This is evidenced by the development of true latency, caseous granulomas and differences in the response to TNF blockade [9], [20]. While the sets of differentially expressed genes in both mice and macaques showed over-representation of GO categories related to the immune response, and similar sets of cytokines were induced in both species, the gene expression profiles were quite distinct. Most of the genes DE in Mtb-infected murine AM were unchanged in Mtb-infected macaque AM and some genes exhibited opposite patterns of expression. The same was true when we compared the expression patterns of DE genes in Mtb-infected macaque AM with the same genes in Mtb-infected murine AM. These results suggest that caution should be employed when extrapolating data from murine models of Mtb to the human situation, although data from humans must be generated to demonstrate which model more closely reflects initial encounters with Mtb by human AM.

Examination of the DE genes following Mtb infection did however reveal a core set of immune response genes that were upregulated in both murine and macaque AM. In contrast to Schu S4 Mtb replicates more slowly with a doubling time in macrophages of approximately 24 hours [37]. Thus, the more robust response seen to Mtb could reflect differences in the kinetics of bacterial growth. There were a total of 91 genes that were DE in both species and 47 of these were upregulated across all infections. The 91 DE genes is a rather conservative estimate because of the strict thresholds used. Pathway analysis of these 47 genes revealed that they mostly belonged to the response to virus pathway and included such genes as IFN-β, IRF7, mx2, RSAD2, CCL5, CXCL10 and OAS2. This may indicate that many viruses, including influenza virus, have developed ways to antagonize these pathways to increase infectivity, whereas Mtb does not employ this technique. Previous studies have indicated that Mtb infected macrophages do activate the interferon pathway [38]–[40]. However, recent studies suggest that activation of this pathway could be beneficial to Mtb pathogenesis [41], [42]. In one study intranasal treatment of mice with a TLR3 agonist prior to and during Mtb infection led to increased severity of disease and failure to control bacterial growth [41]. A study of blood transcriptional signatures in human patients with latent or active TB revealed that an interferon signature was associated with active TB [42] Thus the activation of this pathway by Mtb infection may be a mechanism that allows the bacteria to better survive in the AM.

### Conclusions

Our analysis of AM infection with a variety of pathogens in two species identified both similarities and differences in the way these species responds to the infection. We identified roughly 50 genes that participate in the response to all pathogens we tested in both species. These genes are enriched for several immune response categories and likely represent a core immune response group. In addition, we demonstrated the similarities across species in the way these innate immune cells respond to lethal versus non-lethal pathogens. PR8 influenza and Schu S4, both lethal pathogens in mice, failed to induce a response in AM. In contrast, both species displayed a robust response to Mtb infection. On the other hand our results identified several species and pathogen specific response patterns indicating that care should be taken when using a murine model to study human diseases. Overall our data provides new insights into mechanisms by which the innate immune system responds, and interacts with, invading pathogens.

## Materials and Methods

### Ethics statement

This study was carried out in strict accordance with the recommendations in accordance with standards established in the Animal Welfare Act and the Guide for the Care and Use of Laboratory Animals of the National Institutes of Health. All mice were housed in a specific pathogen-free AAALAC-certified facility at the University of Pittsburgh and were treated under protocols approved by the Institutional Animal Care and Use Committee of the University of Pittsburgh (Assurance Number: A3187-01).

All experimental manipulations and protocols involving macaques were approved by the University of Pittsburgh School of Medicine Institutional Animal Care and Use Committee (Assurance Number: A3187-01). The animals were housed and maintained in accordance with standards established in the recommendations of the Weatherall report, “The use of non-human primates in research”. Non-human primates were housed in caging that conforms to the standards set out in the NIH Guide for the Care and Use of Laboratory Animals in a biosafety level (BSL)2 facility. The animals were provided enhanced enrichment, and frequent monitoring was performed by our trained staff and veterinarians. For bronchoalveolar lavage (BAL), animals were sedated by ketamine prior to each procedure, cetacaine and lidocaine was used prior to lavage to minimize any discomfort, and all animals were monitored carefully during and after the procedure until fully awake. No animal appeared to be in distress from the procedures, as judged by behaviors, appetite, breathing, and movements. Clinical monitoring was also performed on a regular basis, and all monkeys were maintained in good health.

### Animals and BAL isolation

C57BL/6 female mice (6–8 week old) were purchased from Jackson Laboratory (Bar Harbor, ME). Mice were anesthetized and AM were harvested from the lungs of these mice by BAL with 10–12 mls of PBS. The cells were washed in PBS and placed in culture at 1×10^6^ cells/ml in 24 well plates. The purity of the cells was determined by flow cytometry and routinely was >98% macrophage.

Four cynomolgus macaques (*Macacca fascularis*) (Valley Biosystems, Inc., West Sacramento, CA) were >4 years of age and >4 kg in weight. Naïve animals were housed in 4.3 ft2 stainless steel cages at the University of Pittsburgh in a BSL2 facility. Cells were obtained from the airways via BAL as previously described [43].

### Bacteria and virus strains

Mtb (Erdman strain; originally from Trudeau Institute, Saranac Lake, NY) was passed through mice and frozen at −80°C in aliquots. A frozen aliquot was thawed in 7H9 media (Difco) at 37°C and 5% CO_2_, grown for 5–7 days and used to infect AM at an MOI of 2. Ft subsp. tularensis Schu S4 (FSC237) was obtained from BEI Resources. Frozen stocks of Schu S4 were streaked on chocolate II agar plates and incubated at 37°C, 5% CO2 for 2 days and used to infect AM at an MOI of 20. Bacterial stocks were quantitated as previously described [44]. All work with Schu S4 was conducted under BSL3 conditions at the University of Pittsburgh with approval from the Centers for Disease Control and Prevention Select Agent Program. Influenza viruses A/Puerto Rico/8/1934 (H1N1) [45] was adapted to mice through multiple passages. A/Fujian/411/2002 (H3N2) was isolated from a human patient in 2002. To generate viral stocks, specified pathogen free 10 day embryonated hen's eggs (Charles River, Wilmington, MA USA) were infected individually with 200 µL A/PR/8/34 at 10^−2^ and 10^−3^ dilution and incubated at 37°C for 72 hrs. Allantoic fluid was harvested and stored at −80°C. A series of 2-fold dilutions of influenza virus in PBS was prepared and incubated at 25°C for 30 minutes with 50 µl of 0.5% turkey red blood cells (Lampire Biologicals, Pipersville, PA, USA). The extent of hemagglutination was inspected visually, and the highest dilution capable of agglutinating turkey red blood cells was determined. Virus was used at a dilution of 8HA units for stimulation of isolated alveolar macrophages.

### AM infection and sample collection

Harvested AM from mice or macaque were allowed to adhere to the 24 well plates for 30 minutes following which the cells were exposed to various TLR ligands and infectious organisms. Mouse and macaque AM were cultured in RPMI/10% FBS (Gemini Bio-Products, West Sacramento, CA)/1% L glutamine/1% HEPES and RPMI/10% human AB serum (Gemini Bio-Products)/1% L glutamine/1% HEPES respectively. Influenza viruses were administered in a volume of 50 µl of PBS at a concentration of 1×10^5^ pfu/ml. An aliquot of Mtb culture (Day 5, 6, or 7) was washed with DMEM at 1700 rpm for 7 min. Mtb was suspended in BAL media and sonicated using a Virsonic probe sonicator. Mtb was added to AM at a moi of 2. Schu S4 bacteria were used to infect AM at an MOI of 20. Supernatants and cells were harvested at 0, 1, 2, 6, 12, and 24 hours post infection. Supernatants were filtered and then stored at −80° C until they were assayed for cytokine production. Supernatants from cultures infected with Schu S4 were filtered and treated with gentamicin prior to removal from ABSL3 conditions. The cells were collected and immediately placed in TriReagent (Molecular Research Center, Inc. Cincinnati, OH) and stored at −80°C until RNA was extracted.

### Cell staining and flow cytometry

Cell surface staining was performed in PBS containing 2% FBS, with the following antibodies: anti-CD11c-PE, anti-CD11b-FITC, anti-F4/80-FITC, anti-CD3-FITC, anti-CD80-FITC, anti-CD86-FITC (BD Bioscience). For staining cynomolgus macaque BAL, cells were washed in PBS containing 2% BSA, and incubated with the following antibodies: anti-CD11c-PE, anti-CD11b-APC, anti-CD20-APC, and anti-CD3-FITC (BD Biosciences, San Jose, CA). Stained cells were analyzed on LSR II flow cytometer (BD Bioscience) using Flowjo software (Tree Star Inc.).

### Analysis of cytokine production

Mouse BAL culture supernatants harvested following infection were assayed for the presence of seventeen cytokines/chemokines (IL-1β, IL-1α, IL-2, IL-4, IL-6, IL-9, IL-10, IL-12p40, IL-2p70, IL-13, IL-17, TNF-α, IFN-γ, KC, MIP-1β, MCP-1 and RANTES) using a mouse-specific Milliplex Luminex kit (Millipore, Billerica, MA), according to the manufacturer's instructions. Culture supernatants collected from cynomolgus macaque BAL were analyzed for six cytokines/chemokines (IL-1β, IL-6, IL-8, IL-12p40, MIP-1α, TNF-α) using a macaque-specific Multiplex Luminex kit (Millipore) according to the manufacturer's instructions.

### RNA Isolation

RNA was extracted from samples stored at −80°C as previously described [19]. Briefly, 0.3 ml chloroform was added to the TriReagent lysate, vortexed, and incubated at room temperature for 10 min. Samples were transferred to Phase Lock Gel Heavy tubes (Eppendorf/5 PRIME) and centrifuged for 2 min. at 12,000 g. The aqueous phase was transferred to a new tube and an equal volume of isopropanol with 40 µg/ml glycogen (Roche) was added. The samples were incubated overnight at −80°C. Samples were thawed and centrifuged at 4°C for 30 min at 12,000 g. The supernatant was carefully removed and the RNA pellet was washed twice with cold 80% ethanol. RNA pellets were air-dried for 5–10 min. and then resuspended in 15 µl of nuclease free water. RNA quality was determined using an Agilent 2100 Bioanalyzer and quantity was determined by OD_260_ measurements using a DU800 Beckman Coulter Spectrophotometer.

### Microarray Preparation and Processing

Three hundred to 500 ng of total RNA per sample were labeled for microarray using the Low RNA Input Linear Amplification Kit PLUS, one-color kit (Agilent, Santa Clara, CA) following the manufacturer's protocol. cRNA labeled with Cy3 was hybridized to 4×44K whole genome microarrays (Agilent) for 17–19 hrs and washed according to the manufacturer's protocol. Arrays were scanned using the Agilent G2505B microarray scanner and data was extracted using Feature Extraction software version 9.1 (Agilent).

### Array Design and Comparative Expression Profiling Experiments

Mapping the global transcriptional program across macaques and mice was performed with both catalog and custom microarrays designed on the Agilent platform. Whole genome mouse “catalog” arrays generated by Agilent (G4122F) were used for mouse samples. In some experiments the identical oligonucleotide probe set for mouse genes was used with a different arrangement on the array (“custom”). The Agilent eArray platform was used to design custom 4×44K whole genome macaque arrays based on *Macaca mulatta* sequence data available in the NCBI database on 3/23/2007. Reference sequences with verified, confirmed, and provisional status were incorporated. Annotation of the rhesus entries was supplemented using human genes based on sequence homology.

### Identifying differentially expressed genes

Array data used in this study can be accessed at EBI Array Express, Accession number; E-MTAB-427. Normalization was done using d-chip using the median array of time point zero as the reference after applying log_2_ transformation to all values. Replicates in each time point were merged by taking the median over all replicates in that time point, that were not filtered out due to extreme values. Time points that showed replicate fluctuation of more than 99% quantile of the array or were spiking compared to the rest of the time series were removed and estimated using KNNimpute [46]. Genes with more than two time points removed were discarded completely. Since we had generated a large number of time point 0 arrays we generated a single set of values for this time point for each of these species by taking the median value for the 33 mouse and 15 monkey replicates at this time point.

### Dendrogram analysis

The dendrograms were produced using hierarchical clustering of differentially expressed monkey and mouse genes. At each time point, replicates were collapsed into a single value. Then the median value of all nonzero time points of each DE gene was used to generate an inter-disease distance matrix using 1- Spearman correlation as the distance metric. Hierarchical clustering was then performed using average linkage. To obtain a measure of confidence for each branch in the dendrogram, we randomly permuted the gene expression values within each disease and produce corresponding dendrograms as described above. Among the 1000 permuted dendrograms, we counted how many times a particular clade was observed and recorded its frequency. This frequency reflects how frequently we were to observe such node structure by random, and thus provides a measure of confidence for each branch within the dendrogram.

### Soft clustering analysis

Due to factors such as measurement error and ambiguity of cluster boundaries, we found that available clustering methods led to situations in which orthologous genes with similar expression patterns could be misplaced into different clusters. Accordingly, we used a “soft” clustering approach that integrates expression profiles with gene sequence orthology in a modified *k*-means model. This algorithm includes an adjustable weight that rewards ortholog co-clustering. Unlike standard clustering methods, which focus solely on cluster coherence, the soft clustering method can simultaneously detect both similar and divergent behavior between orthologs. When orthologs are not co-clustered despite the addition of a reward, we can be assured that their dynamic profiles truly differ. The reward weight *W* and the number of clusters *k* were scanned over a range of values [0,0.5] and [10], [30] respectively, over 50 random start repeats. Intermediate values of *W* = 0.3 and *k* = 20 were shown to have a minimal increase in within-cluster variance while maximizing the enrichment of GO annotations for immune related terms.

### Reconstructing dynamic networks using DREM

The Dynamic Regulatory Evens Miner (DREM) [24], [47] uses Input-Output Hidden Markov Models (IOHMMs) to integrate expression and motif data to reconstruct dynamic regulatory networks. Expression data is used as the temporal input to the IOHMM and binding predictions based on motif data [48] are used as the additional static input information. Thus, the model generated by DREM encodes a mapping from motif data to the observed temporal expression values. The resulting set of hidden states and transitions between them leads to a global dynamic map. Each gene is then assigned to a specific path in the map based on its time series expression data and the set of motifs identified in its promoter region. Following this assignment we compute association scores for TFs (that bind to the identified motifs) and splits using the hypergeometric distribution enrichment calculation.

## Supporting Information

Figure S1 **Alveolar macrophages express a distinct pattern of response to TLR ligands**. (A). Murine AM were exposed to a series of TLR ligands to characterize their responsiveness to defined stimuli. After 18 hours of stimulation by LPS, CpG or poly I∶C to engage TLR4, TLR9 and TLR3 respectively, cells were examined for changes in phenotype. (B) Poly I∶C induced a robust up-regulation and LPS a more moderate up-regulation of CD86. There was no change in CD80 cell surface expression and only a slight increase in MHC class II following exposure to poly I∶C or LPS. CD11b expression increased on the cell surface following exposure to media alone or CpG, and was unaltered when cells were exposed to LPS or poly I∶C. (C, D) Luminex analysis of supernatants (SN) from these cultures revealed three main patterns of cytokine production: cytokines induced by LPS alone, such as IL-6, TNF and CXCL1 (KC); cytokines induced by poly I∶C alone, such as IFN-β, CXCL10 (IP-10) and CCL5 (RANTES); and those induced by both LPS and poly I∶C, such as CCL3 (MIP-α) and CCL4 (MIP-β). This pattern of cytokine production is indicative of activation of the MyD88/NF-κB (LPS), TRIF/IRF3 (LPS and poly I∶C) and TRIF/IRF7 (poly I∶C) pathways in these cells. No cytokines were detected following exposure to medium alone or CpG, consistent with the observation that murine AM do not express TLR9.
(TIF)

Figure S2 **Murine AM respond to TLR4 and TL3 but not TLR9 ligands.** AM were isolated from C557BL/6 mice by bronchoalveolar lavage as described in the Methods. The cells were cultured in the presence of medium, poly I∶C (10 µg/ml), LPS (100 µg/ml) or CpG (10 µg/ml) for 18–20 hours after which cells were analyzed by flow cytometry and supernatants were collected for Luminex analysis. (A) Giemsa stain of murine BAL following cytospin demonstrating a macrophage-like morphology, and a high degree of purity. (B) Flow cytometric analysis of murine AM exposed to medium (light blue), poly I∶C (brown), LPS (green) or CpG (dark blue) for the expression of CD11b, CD86, MHC class II and CD80. The red line represents the isotype control and the results shown are representative of three independent experiments. (C) Cytokines and chemokines were detected in the supernatants of AM treated with the indicated TLR ligands using Luminex technology. (D) The presence of IFN-β in the same supernatants was determined by ELISA and was only detected when cells were exposed to poly I∶C. Results shown in C and D represent the mean ± SEM of 3–4 independent experiments. Statistics were performed using one way ANOVA with a Tukey post test. P values ** <0.01, *** <0.001.
(TIF)

Figure S3 **Murine and macaque AM show distinctive responses to bacteria and virus.** Cytokines were detected as early as 2 hours post infection and these peaked at 6 hours and were maintained at the same levels until 24 hours post infection. (A) In mice, significant levels of TNF and IL-1 were observed following infection with Mtb and Fuj/02 influenza, whereas no cytokines were detected following exposure to PR8 or Schu S4. (B) In macaques, Mtb and PR8 influenza induced strong TNF production, whereas Schu S4 induced IL-1.
(TIF)

Table S1 **Number of differentially expressed genes identified for each pathogen and species.**
(DOCX)

Table S2 **Expression values of DE genes from Macaque arrays.** Values are log_2_ transformed and represent the fold change from time = 0.
(XLSX)

Table S3 **Expression values of DE genes from Murine arrays.** Values are log_2_ transformed and represent the fold change from time = 0.
(XLSX)
