# Supplementary material for: Large Scale Comparison of Innate Responses to Viral and Bacterial Pathogens in Mouse and Macaque
Source: PLoS One. 2011 Jul 18;6(7):e22401. doi: 10.1371/journal.pone.0022401 (PMC3138787; doi:10.1371/journal.pone.0022401)
Supplement: Table S1 — Number of differentially expressed genes identified for each pathogen and species. (DOCX) [file pone.0022401.s004.docx]

### Supplementary Table 1. Number of differentially expressed genes identified for each pathogen and species.

|  | Mock | PR8 | Fuj/02 | Mtb | Schu S4 | Total |
| --- | --- | --- | --- | --- | --- | --- |
| Macaque | 751 | 608 | 362 | 695 | 678 | 1387 |
| Mouse | 461 | 560 | 692 | 429 | 714 | 1383 |

### **Supplementary Figures**

###
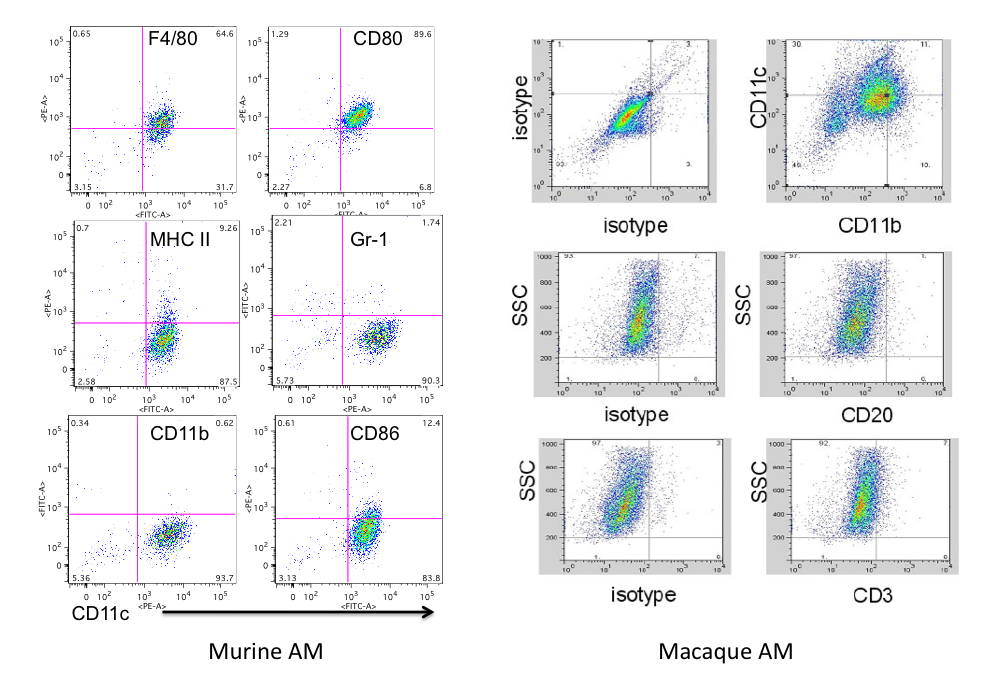


### Supplementary Figure 1.

###
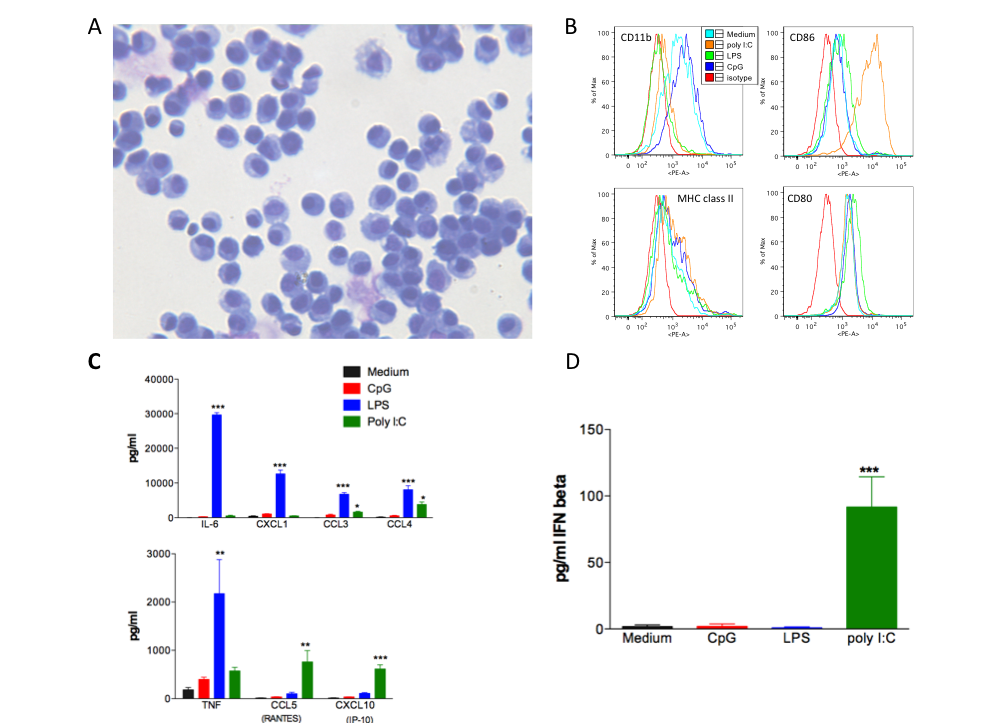


### Supplementary Figure 2.

###
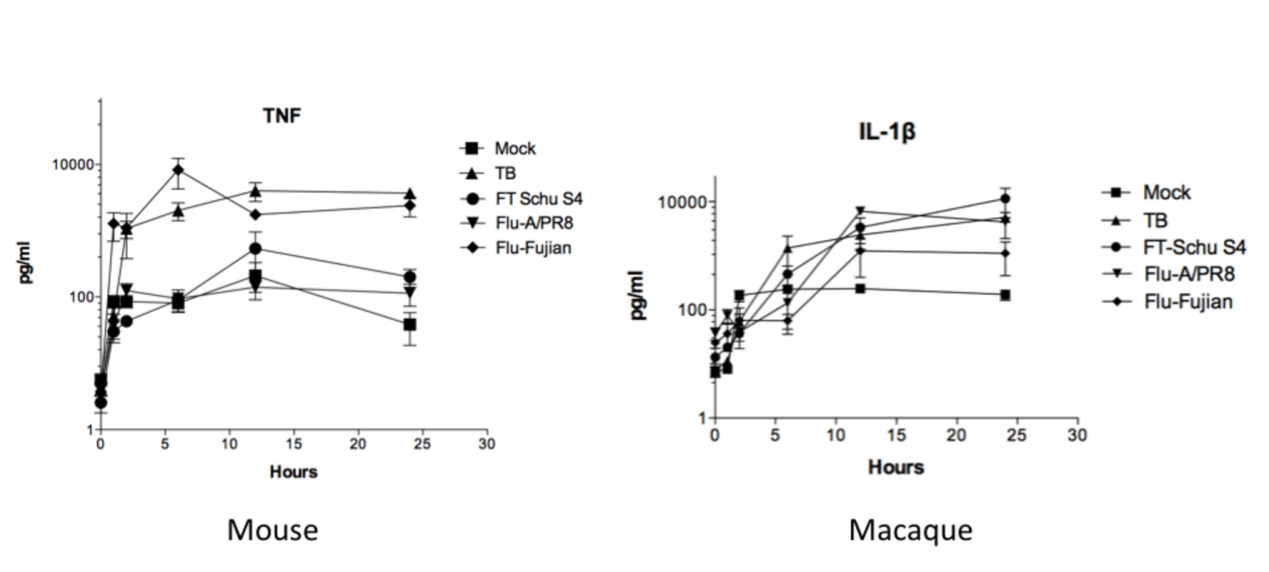


### Supplementary Figure 3.
